# Simulation of the Long-Term Toxicity Towards Bobwhite Quail (*Colinus virginianus*) by the Monte Carlo Method

**DOI:** 10.3390/jox15010003

**Published:** 2024-12-26

**Authors:** Nadia Iovine, Alla P. Toropova, Andrey A. Toropov, Alessandra Roncaglioni, Emilio Benfenati

**Affiliations:** Department of Environmental, Health Science, Istituto di Ricerche Farmacologiche Mario Negri IRCCS, Via Mario Negri 2, 20156 Milano, Italy; nadia.iovine@marionegri.it (N.I.); andrey.toropov@marionegri.it (A.A.T.); alessandra.roncaglioni@marionegri.it (A.R.); emilio.benfenati@marionegri.it (E.B.)

**Keywords:** QSAR, NOEL, NOEC, Bobwhite quail, Monte Carlo method, CORAL software

## Abstract

In this study, models for NOEL (No Observed Effect Level) and NOEC (No Observed Effect Concentration) related to long-term/reproduction toxicity of various organic pesticides are built up, evaluated, and compared with similar models proposed in the literature. The data have been obtained from the EFSA OpenFoodTox database, collecting only data for the Bobwhite quail (*Colinus virginianus)*. Models have been developed using the CORAL-2023 program, which can be used to develop quantitative structure–property/activity relationships (QSPRs/QSARs) and the Monte Carlo method for the optimization of the model. The software provided a model which may be considered useful for the practice. The determination coefficient of the best models for the external validation set was 0.665.

## 1. Introduction

Agricultural and industrial activities rely heavily on the widespread use of chemicals, which has resulted in significant environmental contamination [1,2]. The ongoing rise in chemical pollution underscores the need for comprehensive hazard and risk assessments to prevent adverse effects on various non-target organisms, which are easily exposed to pollutants such as pesticides. Avian species can be exposed to harmful chemicals through several routes: ingestion, inhalation and dermal contact [3]. These exposure pathways can lead to severe toxicological effects, impairing reproduction and survival, and potentially causing population declines that disrupt local ecosystems. Understanding the avian toxicity of various chemicals is critical for protecting bird species and maintaining healthy ecosystems. Ecological risk assessment plays a pivotal role in evaluating the potential side effects of these chemicals on non-target species, thereby helping to preserve ecological balance [4,5].

Birds are essential to both ecosystems and human society due to seed dispersers, and natural pest controllers, which contribute to biodiversity, food production, and agricultural sustainability. They help maintain ecosystem health by regulating insect populations and supporting plant reproduction. Additionally, birds serve as environmental indicators, reflecting changes in habitat quality and potential risks from toxins. Protecting birds and their habitats is crucial for ecological balance and human well-being [6,7].

The reproductive health of birds is especially vulnerable to long-term exposure to chemicals, as they can interfere with hormone systems, impair reproductive success, and cause long-term population declines [8,9,10,11]. In this context, evaluating the long-term toxicity (LTT) and reproductive impacts of pesticides is essential to protect bird species from adverse effects.

To assess the toxicity of chemicals in birds, the Organisation for Economic Co-operation and Development (OECD) has developed a series of standardized test guidelines (TGs), such as the TG 206 (1984) for Reproduction Toxicity, TG 223 (2016) for Acute Oral Toxicity, and TG 205 (1984) for Dietary Toxicity [12,13,14]. The assessment for avian reproduction toxicity (which involves LTT) is usually conducted using either the Northern Bobwhite quail (*Colinus virginianus*), mallard duck (*Anas platyrhynchos*) or Japanese quail (*Coturnix japonica*) as indicator species. Standardized endpoints, such as the No Observed Effect Level (NOEL) and No Observed Effect Concentration (NOEC), play a crucial role in risk assessment, providing key measures of chemical safety for non-target species. NOEL refers to the highest concentration of a substance at which no adverse effects are observed, while NOEC is the concentration at which no significant effects on a population are detected. These endpoints are valuable for assessing the LTT in non-target organisms such as birds and are widely used in ecological risk assessments to inform regulatory decisions [4,15].

The experimental definition of toxicity is expensive and time-consuming. Furthermore, laboratory animals are required, with the related ethical issues [5,16]. For these reasons, the use of in silico methods has been proposed in several studies and can be helpful in assessing a large number of endpoints [17,18,19,20,21,22]. In this paper, we described the data collection and the quantitative structure-activity relationship (QSAR) approach used to develop a model for the LTT of avian. The model is based on the correlation weights of molecular features used to calculate the descriptor within the CORAL software (http://www.insilico.eu/coral/, accessed 24 December 2024). The approach considered here has been applied to a wide variety of problems [23,24].

## 2. Materials and Methods

### 2.1. Data

Here, we considered the NOEL and NOEC for Bobwhite quail. The molecular structure is represented by the simplified molecular input line entry system (SMILES) [25]. This format is one of the most widely used, because it is compact, and uses symbols related to the atoms present in the molecule, indicating the bonds and ramifications. An example is given in Table 1. SMILES generated by VEGAHUB software were considered here (https://www.vegahub.eu/download, accessed 24 December 2024).

Experimental values were collected from the EFSA OpenFoodTox database version 5 (released in October 2023). Data for NOEC and NOEL (mg/kg bw/day) related to LTT or reproduction toxicity in Bobwhite quail were collected. The data collected were analysed to identify possible duplicates. If a compound had several data points, the most toxic one, in our case the one with the lowest value, was kept. Pruning spurious information led to a dataset of 139 experimental values for pesticides. These were converted to the logarithmic scale.

The set of all compounds was split into: (i) the active training set (≈25%), (ii) the passive training set (≈25%), (iii) the calibration set (≈25%), and (iv) the validation set (≈25%). Each set has a defined task. The active training set is used to build up the preliminary model: molecular features extracted from the SMILES of the substances in the active training set are involved in the Monte Carlo optimization using the CORAL software (http://www.insilico.eu/coral/, accessed 24 December 2024), which provides correlation weights for the above features. The CORAL software simply uses the SMILES of the molecules to build up the model, and the descriptors are parts of the SMILES. Conversely, most of the other in silico models have to generate molecular descriptors to be used as input for the model. Thus, CORAL software is easier, and as a result, it directly identifies the atoms and bonds in the molecule associated with the effect. The correlation weights defined by CORAL software give the maximal target function (*TF*), calculated using descriptors (the sum of the correlation weights) and endpoint on the active training set. The passive training set has to check whether the model for the active training set is satisfactory for SMILES that were not involved in the active training set. Since there are several parameters to be defined, the calibration set is used to detect the start of overtraining (overfitting). At the beginning of the optimization, the correlation coefficients between experimental values of the endpoint and the descriptor simultaneously increase for all sets. When the correlation coefficient for the calibration set reaches the maximum (this is the start of the overtraining), further attempts at optimization lead to a decrease in the correlation coefficient for the calibration set. Optimization should be stopped when overtraining starts. At this point, the model development is complete, and the model selected using the results of the calibration set should be considered the final one; the statistics on the calibration set should represent the values of the model selected. Once the Monte Carlo optimization procedure is complete, the validation set is used to assess the predictive potential of the model on substances which have not been used in the model development. Five random splits obtained as above are considered here.

### 2.2. Optimal Descriptors

Modelling based on optimal descriptors involves several levels. The first one is aimed to identify the list of structural parameters of the molecules of the training set. The second level addresses the threshold to establish the list of attributes not used by the model and the list of active SMILES attributes. The third level is used to identify the list of parameters that are apparent promoters of increase or apparent promoters of decrease of the endpoint.

The selected list of molecular features extracted from the database involves the so-called SMILES atoms, which are one symbol or a group of symbols that cannot be considered separately [26]. Fragments of local symmetry (FLS) are the second category of molecular features available from SMILES notations [26]. These are compositions in the forms of XYX, XYYX, and XYZYX, where X, Y, and Z are arbitrary SMILES atoms. Thus, they take into consideration a local, focused moiety of the molecule and not the global symmetry. FLSs use SMILES-atoms containing only one character.

The optimal descriptors (DCW) considered here are calculated as follows:

(1) $$DCW\left(T,N\right)=\sum CW\left(S_{k}\right)+\sum CW\left(SS_{k}\right)+CW\left(XYX\right)+CW\left(XYYX\right)+CW\left(XYZYX\right)$$

*CW*(*x*) are correlation weights for molecular features extracted from SMILES. *S_k_* is a SMILES atom; *SS_k_* is a pair of SMILES atoms that are neighbors in the SMILES line. Table 1 sets out the definition of fragments of local symmetry in SMILES. For the FLS of the format XYX in our case there are four sequences, and two sequences appear for the format XYZYX, while the sequence XYYX is not represented in the SMILES. The *T* is the threshold related to the frequency of a SMILES attribute in the active training set. Here *T* = 1 is used. In other words, if a SMILES attribute occurs even once in the active training set, it is considered an active one, and conversely, if it is absent in the active training set, then it is not used. However, from a statistical point of view, it is preferable to use attributes that appear in the active training set as many times as possible. The *N* is the number of epochs of the Monte Carlo optimization. One epoch indicates the modification of correlation weights of all active SMILES attributes. Here *N* = 15 is used. Thus, below in this work, the *DCW* is defined as *DCW (1, 15)*.

### 2.3. Optimization of Correlation Weights

Monte Carlo optimization is the process of the maximization for a target function, which is in fact a mathematical function of many parameters including the step of dividing the available data into four sets: active training, passive training, calibration, and validation sets. The task of the active training set is to identify suitable correlation weights for a model/correlation. The task of the passive training set is to verify whether the correlation weights are available for similar substances. The calibration set is aimed at double-checking the results of the active and passive training sets. Finally, the validation set aims to assess the model’s predictive potential using substances not used to build up the model. The optimization of correlation weights with the target function is carried out by gradual replacement of their initial values using the Monte Carlo algorithm.

**Figure 1:**
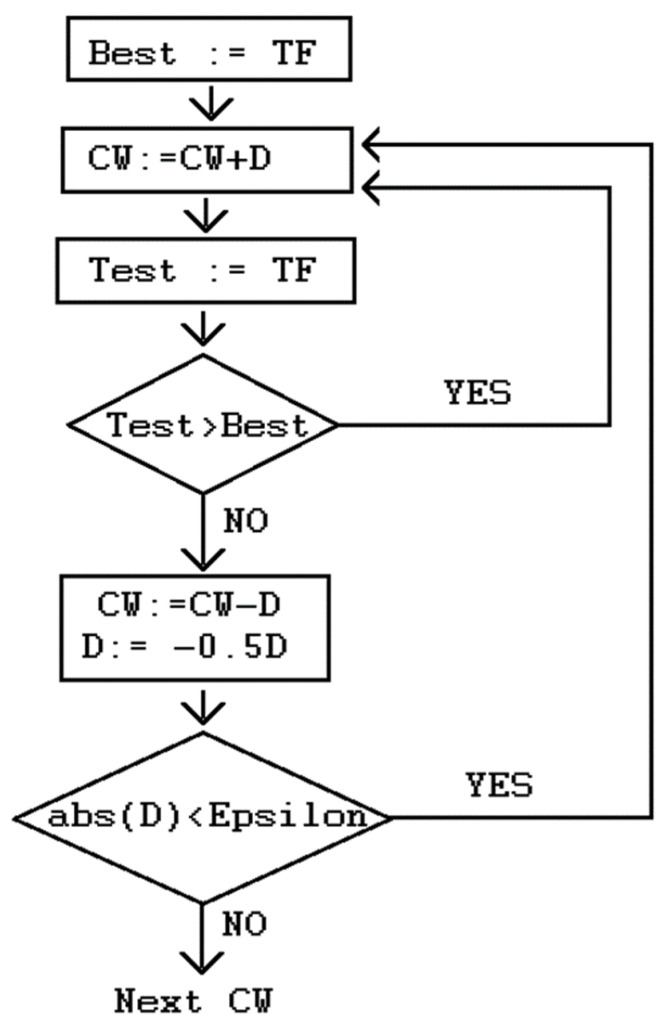
The flow chart of the optimization of the correlation weight with target function (*TF*). *D* is some delta, i.e., the coefficient for modification of the correlation weights.

The flow chart of the transformation of the correlation weight value is shown in Figure 1.

The optimization process applied here involves special components termed index of ideality of correlation (*IIC*) and correlation intensity index (*CII*) [26]. The *IIC* and *CII* should increase the “system’s attention” to that part of the training set more useful to identify general lessons, not only related to the training set but relevant to the calibration set. The influence of these factors can be regulated by using the weighting coefficients for the *IIC* and *CII*. The selection of these coefficients is carried out empirically, based on the results of preliminary observations of the stochastic optimization system, with different weights for the *IIC* and *CII*.

Thus, the target function applied here is calculated as follows:

(2) $$TF=R_{A}+R_{P}-\left|R_{A}-R_{P}\right|\times 0.1+0.3\times CII+0.5\times IIC$$

*R_A_* and *R_P_* are the determination coefficient values on the active and passive training sets, respectively.

Having the numerical data on the correlation weights, one can calculate the LTT towards quail with Equation (3):

(3) $$LTT=C_{0}+C_{1}\times DCW\left(1,15\right)\;$$

*C*_0_ and *C*_1_ are regression coefficients. The *DCW*(1,15) is the optimal descriptor calculated with Equation (1).

Table 2 contains an example of the calculation of the *DCW*(1,15) for the calculation weights of SMILES attributes obtained for split #1.

## 3. Results

Attempts to build a model without FLS correlation weights gave the results reported in Table 3. Unfortunately, these correlations are rather weak; for instance, the *R^2^* of the validation set rarely reaches 0.5. However, the use of correlation weights of the FLS, as will be shown further on, improved the predictive potential of the model.

The statistical characteristics of models obtained with the correlation weights of FLS are better than those without these correlation weights. We replicated the modelling using five splits to give a more robust assessment of the results. The five models for the endpoint calculated with FLS are the following:

split-1: LTT = −1.949(± 0.019) + 0.1307(± 0.0023) * DCW(1,15) (4)

split-2: LTT = −1.867(± 0.026) + 0.1894(± 0.0040) * DCW(1,15)(5)

split-3: LTT = −1.587(± 0.021) + 0.0536(± 0.0059) * DCW(1,15)(6)

split-4: LTT = −1.599(± 0.013) + 0.1637(± 0.0028) * DCW(1,15) (7)

split-5: LTT = −2.013(± 0.020) + 0.1237(± 0.0050) * DCW(1,15) (8)

Table 4 lists the statistical characteristics of the models calculated with Equations (4)–(8). *R^2^* on the validation set are always higher than 0.5 and reaches 0.66 in the third split. Another useful statistical parameter, the mean absolute error (MAE), is in the range of 0.23–0.41. These statistical values are good enough to accept the approach used here, also considering the small number of substances used to build up the model. Furthermore, the endpoint is quite a complex one, since it is related to a number of mechanisms associated with the LTT. This endpoint is probably more difficult to be modelled compared with acute toxicity since it involves a larger set of toxicological processes related to prolonged exposure to the toxic substance. Finally, another element is that the model relates to pesticides, which contain several categories of substances with complex chemical structures.

In addition to the statistical characteristics of the models, a highly desirable addition is the mechanistic interpretation that allows to identify molecular features whose presence in the molecule can contribute to the increase in the magnitude of the considered endpoint, as well as structural features that can contribute to the decrease in the magnitude of the endpoint in question. Table 5 contains data on five Monte Carlo optimization runs of correlation weights of different molecular features extracted from SMILES (split #1, simulation using FLS). It is clear that if a correlation weight is consistently positive for all Monte Carlo optimization runs, then the corresponding molecular structure element should be considered as a promoter of increasing the endpoint under consideration. Conversely, if a correlation weight is consistently negative in a series of optimization runs, then the corresponding structure element should be considered as a promoter of decreasing the endpoint under consideration. One can see, that in addition to “traditional SMILES attributes” which are promoters of increase or decrease, the FLS are present in the lists too.

The applicability domain for these models was determined through the so-called statistical defects of SMILES. According to the above statistical defects, 11, 21, 18, 6, and 12 outliers are observed for splits #1–#5, correspondingly.

Table 6 compares the statistical quality of the models suggested here with others from the literature. Unfortunately, there are only a few works devoted to modeling toxicity to quails, so the collection in Table 6 is scarce. We underline that the endpoint that we address here is related to long-term exposure, and thus this endpoint is different from the endpoints addressed for avian toxicity in the other studies in the literature, related to acute toxicity effect. As we commented, the long-term exposure effects are more complex and difficult to evaluate, but they are of higher ecotoxicological relevance since this endpoint is more representative of the real situation. Since the calibration set represents the proper final set, the statistics on that set should be considered too. However, to be closer to the usual way of representing the results of the training set, we also provide the overall statistics of the calibration set, with the active and passive training sets.

## 4. Discussion

The approach to constructing QSPR/QSAR models considered here is based on the use of so-called optimal descriptors. The idea of optimal descriptors was initially related to the use of molecular graphs. More precisely, it was supposed to use, instead of numerical values of graph invariants, some coefficients that, when transformed into their sum, would give the maximum correlation coefficient with the endpoint under study. In fact, this project can be considered as an extension of the flexible descriptors proposed in [26].

However, if the mentioned work focused on modifying the diagonal of the adjacency matrix of a molecular graph (in other words chemical elements), the proposed extension [28] already concerned the off-diagonal elements of the adjacency matrix, which from a chemical point of view is a prototype of covalent bonds.

One of the important, although not the main, circumstances for developing models in general, and for QSPR/QSAR models in particular, is the convenience of constructing the models of interest. Despite the rapid growth of the possibilities of attracting memory resources and the speed of modern computers, the complexity of implementing modeling in conditions of representing molecules through graphs in the corresponding databases intended to serve as input for constructing models remains inconvenient. When developing models, it is much more convenient to use databases where molecules are presented in a more compact form, containing as much relevant information as possible, which can be sorted if desired and, if necessary, shortened.

There are several options for representing the molecular structure that to some extent satisfy the above-mentioned attractive and useful possibilities when applied to QSPR/QSAR analysis. Common molecular representations suitable for the above application are SMILES [26] and InChI [29]. The International Chemical Identifier notation system (InChI) is an alternative to the SMILES molecular representation system. The information content of INCHI is much greater than that of SMILES. However, extracting this information without special software is difficult for the common user.

In relation to InChI, it can be said that although the length of the string InChI is much greater than SMILES, nevertheless it is a compact representation capable of serving as a basis for developing and using databases capable of being input for QSPR/QSAR analysis. However, the investigation of information presented by InChI is much less convenient than SMILES, from the point of view of the human user. Like a SMILES notation, an InChI string is derived from a molecular structure representation. However, InChI is intended for computer use. It is typically derived from structure representations by software, whereas SMILES supports molecular communication between humans and computers [30].

Indeed, the CORAL software was used in the SMILES format quite often for a wide variety of physicochemical properties and biological activities such as toxicity of inorganic compounds [31], cellular activity induced by nanomaterials [32], chromatography retention indices of volatile organic compounds [33], binding affinity of endocrine disruptor [34], search for radiopharmaceutical agents [35], novel inhibitors against pancreatic cancer [36], influenza inhibitors [24], predicting the permeability of drugs [37], search for anti-prostate cancer agents [38], psychoactive drugs [39], simulation of drug-induced nephrotoxicity [40] whereas InChI did not find wide application for the development of optimal descriptors [29].

From a philosophical point of view, the concept of a “dialectical pair” is often used, implying a pair of concepts capable of directing thought in some rational constructive direction, in particular for the formation of useful hypotheses. Examples of dialectical pairs are essence and phenomenon; quality and quantity; space and time; cause and effect; necessity and chance; reality and possibility; matter and consciousness; and object and subject. Within the same perspective, optimal descriptors lead to the formulation of this dialectical pair: randomness and prediction.

It is obvious that the stochastic processes are related to randomness. However, due to the objective functions used in the Monte Carlo optimization processes, randomness becomes capable of creating a prediction based on the information provided by SMILES. In our experience, it is important to use the same algorithm to write the SMILES and the same should be used to generate the SMILES in the external verification.

As noted above, FLS proved to increase the predictivity of the models. We can only formulate a hypothesis regarding the reason for this. A possible explanation is that these fragments identify the fact that in a certain part of the molecule, there is a duality of circumstances, and the same phenomenon, e.g., the initial step generating the toxic effect, can occur in two locations, and this represents an increase in the probability that the event occurs. The identification of this case, offered by the FLS, improves the description of the molecular features on the basis of the adverse effect, probably.

The computer experiments conducted have shown that the FLS can help improve the predictive potential of models. Following the principle of “QSPR/QSAR are random event” [41], any conclusions related to QSPR/QSAR analysis must be confirmed based on the observation of not one, but several distributions of training and validation samples. Regarding the thesis that FLS can be useful as a tool for improving the predictive potential, it can be stated that this is the case for several (five) splits into a training set and a validation set.

While recognizing the shortcomings of the current version of fragments of local symmetry, it is also necessary to note that, in principle, more rigorous versions of the system of FLS can be formulated and developed. In particular, it is possible to specify FLS not only by symbols but also by chemical elements. On the other side, it is possible to remove from consideration such versions of local symmetry fragments that include obviously dubious symbols, such as brackets, numbers, and others (Table S1).

## 5. Conclusions

In this paper, we introduced QSAR models for long-term toxicity towards birds. The data has been obtained from the EFSA database OpenFoodTox, which represents an authoritative source. The CORAL software with Monte Carlo optimization has been used. In this way, optimal descriptors provided models characterized by satisfactory predictive potential for long-term toxicity towards quail. This is the first model, at the best of our knowledge, for this important kind of endpoint, since it is related to effects which may occur in real conditions, considering prolonged exposure. Other models, previously published, also by our laboratory, addressed acute toxicity towards birds, which is a particular case of effect, easier to be modelled, but less relevant. The predictive potential was estimated based on the results of constructing five models using significantly different partitions into training and validation sets. Structured training sets were used. They consisted of three groups of compounds, the so-called active training, passive training, and calibration sets. The observed average value of the determination coefficient on the validation set for five computer experiments of constructing toxicity models is 0.57±0.05. Semi-quantitative molecular features conveyed through the fragments of local symmetry tested here may be useful for developing models of the endpoint considered. The contribution of the index of ideality of correlation in the stochastic process of the Monte Carlo method optimization is quite valuable.

These models will be implemented in the VEGAHUB website (www.vegahub.eu), for free use.

## Figures and Tables

**Table 1:** An example of formulation of fragments of local symmetry (FLS) for the SMILES of view O=C(O)c1nc(c(c(N)c1Cl)Cl)Cl.

Types of Fragments of Local Symmetry	Codes for Calculation of Optimal Descriptor
**FLS XYX**	
c(c; (c(; c(c; (c(	[xyx4]
**FLS XYYX**	
Absent	[xyyx0]
**FLS XYZYX**	
c(c(c; (c(c(	[xyzyx2]

**Table 2:** An example of calculation of the *DCW*(1,15) that is equal to the sum of all correlation weights for pesticide represented by SMILES of view O=C(O)c1nc(c(c(N)c1Cl)Cl)Cl (the descriptor value is 16.10).

SMILES Attribute	Correlation Weight of SMILES Attribute, *CW(x)*	NA *	NP	NC
**(*S_k_*)**				
O...........	−0.4891	33	31	33
=...........	−0.1378	30	25	34
P...........	2.5439	4	2	1
(...........	−0.2469	34	33	35
O...........	−0.4891	33	31	33
c...........	−0.2147	28	30	30
1...........	0.5435	30	32	32
c...........	−0.2147	28	30	30
c...........	−0.2147	28	30	30
c...........	−0.2147	28	30	30
(...........	−0.2469	34	33	35
c...........	−0.2147	28	30	30
(...........	−0.2469	34	33	35
c...........	−0.2147	28	30	30
1...........	0.5435	30	32	32
(...........	−0.2469	34	33	35
C...........	0.0241	34	33	35
(...........	−0.2469	34	33	35
S...........	0.7864	12	11	11
C...........	0.0241	34	33	35
(...........	−0.2469	34	33	35
(...........	−0.2469	34	33	35
O...........	−0.4891	33	31	33
C...........	0.0241	34	33	35
C...........	0.0241	34	33	35
(...........	−0.2469	34	33	35
N...........	0.7755	25	23	29
C...........	0.0241	34	33	35
(...........	−0.2469	34	33	35
C...........	0.0241	34	33	35
(...........	−0.2469	34	33	35
C...........	0.0241	34	33	35
***SS_k_***				
O...=.......	0.0016	29	24	30
P...=.......	8.2913	1	1	0
P...(.......	2.0343	4	2	1
O...(.......	0.2868	30	23	26
c...O.......	0.7112	7	8	8
c...1.......	0.2000	25	26	30
c...1.......	0.2000	25	26	30
c...c.......	0.0593	28	29	29
c...c.......	0.0593	28	29	29
c...(.......	0.0243	27	28	27
c...(.......	0.0243	27	28	27
c...(.......	0.0243	27	28	27
c...(.......	0.0243	27	28	27
c...1.......	0.2000	25	26	30
1...(.......	1.6836	19	19	11
C...(.......	0.0663	33	32	35
C...(.......	0.0663	33	32	35
S...(.......	1.1459	12	9	8
S...C.......	0.8502	5	5	8
C...(.......	0.0663	33	32	35
(...(.......	−0.5271	22	19	21
O...(.......	0.2868	30	23	26
O...C.......	0.1543	21	22	16
C...C.......	0.7926	19	16	18
C...(.......	0.0663	33	32	35
N...(.......	−0.7782	19	21	22
N...C.......	0.0455	9	13	8
C...(.......	0.0663	33	32	35
C...(.......	0.0663	33	32	35
C...(.......	0.0663	33	32	35
C...(.......	0.0663	33	32	35
***FLS***				
[xyx7]......	−0.8997	6	1	6
[xyyx0].....	0.5900	29	29	24
[xyzyx1]....	0.0854	9	8	8

* NA, NP, and NC are frequencies of SMILES attribute in the active training, passive training, and calibration sets, respectively.

**Table 3:** The statistical characteristics of models of long-term toxicity towards Bobwhite quail built up without correlation weights of FLS.

Split	Set *	*n*	*R^2^*	*CCC*	*IIC*	*CII*	*Q^2^*	*RMSE*	*MAE*	*F*
1	*A*	35	0.4800	0.6486	0.6543	0.7427	0.4215	0.593	0.485	30
	*P*	33	0.7244	0.3748	0.8511	0.8410	0.6951	0.918	0.854	81
	*C*	35	0.5441	0.6699	0.7376	0.7673	0.4501	0.336	0.264	39
	*V*	35	0.3707	-	-	-	-	0.35	0.28	-
2	*A*	34	0.4498	0.6205	0.5962	0.7426	0.3925	0.656	0.538	26
	*P*	35	0.5295	0.4275	0.2649	0.6978	0.3505	0.880	0.783	37
	*C*	35	0.5416	0.6950	0.7357	0.7518	0.5007	0.346	0.286	39
	*V*	34	0.4496	-	-	-	-	0.41	0.34	-
3	*A*	35	0.2803	0.4379	0.5000	0.6605	0.1722	0.640	0.528	13
	*P*	34	0.6210	0.3563	0.7880	0.7937	0.5602	0.965	0.865	52
	*C*	35	0.4920	0.6669	0.7014	0.7643	0.4311	0.399	0.298	32
	*V*	34	0.3430	-	-	-	-	0.46	0.36	-
4	*A*	35	0.7223	0.8387	0.7157	0.8321	0.6912	0.451	0.380	86
	*P*	35	0.4970	0.4180	0.6474	0.6985	0.4453	0.681	0.602	33
	*C*	34	0.3486	0.5869	0.5904	0.7781	0.2470	0.410	0.329	17
	*V*	34	0.5358	-	-	-	-	0.31	0.25	-
5	*A*	34	0.5385	0.7000	0.5793	0.7017	0.4796	0.502	0.422	37
	*P*	35	0.4895	0.5221	0.5927	0.7873	0.4420	0.779	0.722	32
	*C*	35	0.4229	0.6356	0.6503	0.8286	0.3550	0.267	0.236	24
	*V*	34	0.4290	-	-	-	-	0.40	0.32	-

* *A* = active training set; *P* = passive training set; *C* = calibration set; *V* = validation set; *R^2^* = determination coefficient; *CCC* = concordance correlation coefficient; *IIC* = index of ideality of correlation; *CII* = correlation intensity index; *Q^2^* = cross-validated *R^2^*; *RMSE* = root mean squared error; *MAE* = mean absolute error; *F* = Fischer F-ratio.

**Table 4:** Statistical characteristics of models of LTT towards Bobwhite quail built up with correlation weights of FLS.

Split	Set *	*n*	*R^2^*	*CCC*	*IIC*	*CII*	*Q^2^*	*RMSE*	*MAE*	*F*
1	*A*	35	0.6235	0.7681	0.7457	0.7708	0.5877	0.504	0.400	55
	*P*	33	0.7800	0.4614	0.8832	0.8480	0.7527	0.904	0.831	110
	*C*	35	0.6117	0.7652	0.7820	0.7658	0.5512	0.343	0.273	52
	*V*	35	0.5140	-	-	-	-	0.32	0.25	-
2	*A*	34	0.6470	0.7857	0.8044	0.7749	0.6071	0.525	0.418	59
	*P*	35	0.7167	0.5323	0.3230	0.8004	0.6504	0.938	0.816	83
	*C*	35	0.6618	0.7233	0.8135	0.8614	0.6221	0.412	0.311	65
	*V*	34	0.5670	-	-	-	-	0.51	0.41	-
3	*A*	35	0.1421	0.2488	0.2827	0.7475	0	0.699	0.615	5
	*P*	34	0.6175	0.2995	0.7858	0.8005	0.5522	0.892	0.763	52
	*C*	35	0.7052	0.7963	0.8397	0.8397	0.6615	0.233	0.187	79
	*V*	34	0.6650	-	-	-	-	0.28	0.23	-
4	*A*	35	0.7492	0.8567	0.7289	0.8296	0.7199	0.428	0.340	99
	*P*	35	0.7100	0.5958	0.4745	0.7875	0.6818	0.634	0.534	81
	*C*	34	0.4107	0.6308	0.6408	0.8479	0.3130	0.401	0.315	22
	*V*	34	0.5208	-	-	-	-	0.40	0.31	-
5	*A*	34	0.4945	0.6618	0.6251	0.7211	0.4113	0.525	0.455	31
	*P*	35	0.4840	0.4733	0.4878	0.7897	0.4337	0.843	0.752	31
	*C*	35	0.7500	0.8436	0.8658	0.8622	0.7197	0.169	0.142	99
	*V*	34	0.5819	-	-	-	-	0.36	0.27	-

* *A* = active training set; *P* = passive training set; *C* = calibration set; *V* = validation set; *R^2^* = determination coefficient; *CCC* = concordance correlation coefficient; *IIC* = index of ideality of correlation; *CII* = correlation intensity index; *Q^2^* = cross-validated *R^2^*; *RMSE* = root mean squared error; *MAE* = mean absolute error; *F* = Fischer F-ratio.

**Table 5:** Mechanistic interpretation of the model for LTT towards Bobwhite quail (split #1, correlation weights of FLS involved to optimal descriptor calculation).

S or SS or FLS	1	2	3	4	5	NA	NP	NC
Promoters of increase								
[xyyx0].....	1.1230	0.9561	1.0716	1.3799	0.4717	29	29	24
c...2.......	1.9643	1.2383	0.1298	1.6865	1.1871	23	18	18
C...C.......	0.3808	1.2369	1.0098	1.2700	1.1194	19	16	18
S...(.......	0.4506	0.8043	0.1494	0.8849	1.1681	12	9	8
N...C.......	1.3056	1.8532	1.8860	1.3248	1.2236	9	13	8
c...O.......	0.5893	1.2124	0.8237	0.0148	0.7202	7	8	8
n...1.......	0.2268	2.0672	0.4961	1.7561	0.7070	6	4	3
3...(.......	0.4225	0.8864	1.3480	1.5308	0.7303	5	8	4
S...C.......	0.7606	0.9514	1.9550	1.4794	0.5854	5	5	8
P...(.......	2.6666	2.7187	2.8474	3.3673	3.9175	4	2	1
P...........	3.8694	2.9393	2.3262	2.1454	2.8775	4	2	1
[xyzyx2]....	2.2127	1.0702	0.9146	2.0337	2.3060	4	1	3
[xyx4]......	0.5218	1.8897	1.9796	1.3629	1.2598	3	4	3
n...3.......	0.8220	0.7589	0.9489	1.0793	0.7237	3	1	1
S...P.......	3.3034	4.8946	4.4617	2.5290	4.1761	2	1	1
Promoters of decrease								
C...(.......	−0.4395	−0.6585	−0.3236	−0.2932	−0.8184	33	32	35
1...........	−0.6192	−0.4606	−0.0605	−0.1050	−0.4894	30	32	32
=...........	−0.1914	−0.9555	−0.6864	−0.8934	−0.1373	30	25	34
O...=.......	−0.5426	−0.6028	−0.0555	−0.2830	−1.3653	29	24	30
c...........	−0.4842	−0.8222	−0.3887	−0.4997	−0.8995	28	30	30
2...(.......	−1.1851	−1.5421	−1.2887	−1.3368	−1.1119	15	20	13
n...c.......	−0.5251	−1.2618	−0.7232	−0.4177	−0.0434	12	14	8
[xyzyx1]....	−1.9008	−0.4257	−0.3202	−0.6042	−0.0287	9	8	8
n...(.......	−0.7403	−0.6638	−0.4427	−0.8044	−0.6368	7	10	4
N...1.......	−0.0541	−0.8301	−1.0608	−0.0389	−0.7986	6	1	5
[xyx5]......	−2.7243	−3.1202	−1.5869	−1.2596	−1.0190	6	6	4
[xyx7]......	−1.4561	−0.7690	−0.1741	−2.0073	−0.7584	6	1	6
C...3.......	−0.9602	−0.5414	−0.4110	−0.4759	−1.6288	5	5	6
c...N.......	−3.3089	−2.0621	−1.2194	−2.4258	−3.3495	4	9	5
[xyx2]......	−1.9693	−1.3590	−1.3893	−1.3086	−0.4644	4	2	3

**Table 6:** Comparison of different models for toxicity (long-term in our case, acute in the others) toward Bobwhite quail.

Number of Compounds in Training Set	Number of Compounds in Validation Set	R^2^ for Training Set	R^2^ for Validation Set	Reference
41	15	0.67	-	[21]
25	8	0.88	-	[21]
115	32	0.95	0.92	[22]
91	19	0.78	0.65	[27]
104	34	0.49 (0.70 calibration)	0.67	In this work

## Data Availability

Data is contained within the article or Supplementary Material.

## Supplementary Materials

The following supporting information can be downloaded at: https://www.mdpi.com/article/10.3390/jox15010003/s1, Table S1 in Supplementary materials section contains the technical details of the considered models (Split #1).
